# Involvement of the R2R3-MYB transcription factor MYB21 and its homologs in regulating flavonol accumulation in Arabidopsis stamen

**DOI:** 10.1093/jxb/erab156

**Published:** 2021-04-08

**Authors:** Xueying Zhang, Yuqing He, Linying Li, Hongru Liu, Gaojie Hong

**Affiliations:** 1 State Key Laboratory for Managing Biotic and Chemical Threats to the Quality and Safety of Agro-Products, Institute of Virology and Biotechnology, Zhejiang Academy of Agricultural Sciences, 198 Shiqiao Road, Hangzhou 310021, China; 2 National Key Laboratory of Plant Molecular Genetics and National Plant Gene Research Center, CAS Center for Excellence in Molecular Plant Sciences, Shanghai Institute of Plant Physiology and Ecology, Shanghai 200032, China; 3 The James Hutton Institute, UK

**Keywords:** Arabidopsis, flavonol, *FLS1* promoter, MYB21, transcription factors, ROS scavenging

## Abstract

Commonly found flavonols in plants are synthesized from dihydroflavonols by flavonol synthase (FLS). The genome of *Arabidopsis thaliana* contains six *FLS* genes, among which *FLS1* encodes a functional enzyme. Previous work has demonstrated that the R2R3-MYB subgroup 7 transcription factors MYB11, MYB12, and MYB111 redundantly regulate flavonol biosynthesis. However, flavonol accumulation in pollen grains was unaffected in the *myb11myb12myb111* triple mutant. Here we show that MYB21 and its homologs MYB24 and MYB57, which belong to subgroup 19, promote flavonol biosynthesis through regulation of *FLS1* gene expression. We used a combination of genetic and metabolite analysis to identify the role of MYB21 in regulating flavonol biosynthesis through direct binding to the GARE *cis*-element in the *FLS1* promoter. Treatment with kaempferol or overexpression of *FLS1* rescued stamen defects in the *myb21* mutant. We also observed that excess reactive oxygen species (ROS) accumulated in the *myb21* stamen, and that treatment with the ROS inhibitor diphenyleneiodonium chloride partly rescued the reduced fertility of the *myb21* mutant. Furthermore, drought increased ROS abundance and impaired fertility in *myb21*, *myb21myb24myb57*, and *chs*, but not in the wild type or *myb11myb12myb111*, suggesting that pollen-specific flavonol accumulation contributes to drought-induced male fertility by ROS scavenging in Arabidopsis.

## Teaser

MYB21 modulates the accumulation of flavonols through directly binding to the GARE *cis*-element of the *FLS1* promoter, which is essential for the reduced-fertility phenotype of the *myb21* mutant.

## Introduction

Flavonoids are synthesized via the phenylpropanoid pathway and include flavonols, anthocyanins, and proanthocyanidins (Falcone Ferreyra *et al*., 2012). Among them, the colorless flavonols are well known for their physiological functions and serve as protectants against UV radiation, regulators of fertility and auxin transport, and signals for pollinators and other organisms (Mo *et al.*, 1992; Brown *et al.*, 2001; Kuhn *et al.*, 2011; Lewis *et al.*, 2011; Emiliani *et al.*, 2013). In plants, kaempferol, quercetin, and myricetin (and their derivatives) are often the major flavonol components, the composition and distribution of which are influenced by developmental and environmental factors (Winkel-Shirley, 2002).

The biosynthesis of flavonols follows the flavonoid pathway, where at the flavonol branch the oxidation of dihydroflavonols to flavonols is catalyzed by flavonol synthase (FLS) (Wisman *et al.*, 1998; Owens *et al.*, 2008). In *Arabidopsis thaliana*, there are six *FLS* genes, referred to as *FLS1* to *FLS6*. Among their products, FLS1 is functional and shows strong enzymatic activity (Owens *et al.*, 2008; Stracke *et al.*, 2009). FLS3 is responsible for the formation of flavonols in the *fls1* mutant (Preuss *et al.*, 2009). The *FLS1* gene is highly expressed in flowers, which also show flavonol accumulation (Peer *et al.*, 2001; Nguyen *et al.*, 2016). The transcription factors MYB11, MYB12, and MYB111, which belong to subgroup 7 of the R2R3-MYB family, have been reported to regulate flavonol biosynthesis (Stracke *et al*., 2007, 2010). Among them, MYB12 and MYB111 play a major role in the regulation of flavonol biosynthesis. While MYB12 acts mainly in the root, MYB111 acts primarily in the cotyledons. In fact, although flavonol accumulation was drastically reduced in the *myb11myb12myb111* triple mutant, the amounts of flavonol products in pollen grains were equal to that in the wild type (Stracke *et al*., 2007, 2010). This phenotype suggests that there might be additional transcription factors that regulate *FLS1* expression in the stamen.

Many transcription factors have been reported to influence stamen development; among these, MYB21 and its homologs MYB24 and MYB57, which belong to the R2R3-MYB subgroup 19, were either enriched or highly expressed in the stamen (Cheng *et al.*, 2009; Dubos *et al.*, 2010). Mutations of these three MYBs lead to indehiscent anthers, unviable pollen grains, and shorter stamen filaments (Cheng *et al.*, 2009; Song *et al.*, 2011; Qi *et al.*, 2015). It has been demonstrated that gibberellin (GA) induces the expression of *MYB21*, *MYB24*, and *MYB57* by increasing jasmonate production, resulting in the promotion of stamen development (Cheng *et al.*, 2009). Later research revealed that the jasmonate-ZIM domain (JAZ) and DELLA proteins could interact with MYB21 and MYB24 to attenuate their regulatory activity in jasmonate/GA-regulated anther development and filament elongation, respectively (Song *et al.*, 2011; Huang *et al.*, 2020). Most recently, it has been reported that MYB99 modulates phenylpropanoid biosynthesis in a regulatory triad with MYB21 and MYB24 (Battat *et al.*, 2019). Furthermore, Shan *et al.* (2020) reported that FhMYB21L1 and FhMYB21L2 activated *FhFLS1* expression by directly binding to its promoter regions in *Freesia hybrida*. Given that AtMYB21 and AtMYB24 are the homologs of FhMYB21L1 and FhMYB21L2 in Arabidopsis, both have been predicted to regulate *AtFLS1* expression (Shan *et al.*, 2020). However, the molecular mechanism and biological function of MYB21/24/57 in the regulation of flavonol biosynthesis require further investigation *in vitro* and *in vivo*.

Here, we report that MYB21 and its homologs promote the expression of *FLS1* and increase the accumulation of flavonols in Arabidopsis. Furthermore, we show that the representative transcription factor MYB21 regulates *FLS1* expression by directly binding to GARE elements in its promoter, and that Arabidopsis plants overexpressing *MYB21* accumulate flavonols in their flowers. More interestingly, when treated with kaempferol (one of the primary flavonols present in reproductive tissues) or when overexpressing *FLS1*, the siliques of the *myb21* mutant contained more seeds. Consistent with the accumulation of excess reactive oxygen species (ROS) in the *myb21* mutant, treatment with the ROS inhibitor diphenyleneiodonium chloride (DPI) could partly rescue the sterility phenotype of *myb21*. Although the mechanism requires further investigation, we further hypothesized that a MYB21-mediated flavonoid metabolism in pollen grains was essential for stamen development and male fertility, and this is probably because flavonols can prevent ROS from reaching damaging levels.

## Materials and methods

### Plant materials, constructs, and primers

Plants of *Arabidopsis thaliana* ecotype Columbia-0 (Col-0) were grown in a growth room at 22 °C with a 16 h light/8 h dark cycle unless otherwise indicated. The Arabidopsis Genome Initiative identifiers of the three MYBs under study are as follows: *MYB21* (*At3g27810*), *MYB24* (*At5g40350*), and *MYB57* (*At3g01530*). The *myb21* (SALK_042711), *myb24* (SALK_017221), *myb57* (SALK_065776), and *myb21myb24myb57* mutants were kind gifts from D. X. Xie. The seeds of *chs/tt4-11* (SALK_020583) and *myb11myb12my111* mutant were a gift from J. R. Huang.

Transgenic plants overexpressing *MYB21-FLAG* (three copies of FLAG in tandem) in Col-0 and the *myb21* mutant were generated by cloning *MYB21* cDNA into the JW819 vector under the control of the 35S promoter. The overexpression of *MYB21-FLAG* was able to complement the phenotype of the *myb21* mutant, including its shortened stamen and reduced fertility (Supplementary Fig. S1). To produce the promoter–*GUS* reporter construct, the upstream DNA fragments of *FLS1* (837 bp) were amplified by PCR and ligated into the pBI121 vector. β-Glucuronidase (GUS) histochemical analysis was performed as described previously (Jefferson *et al.*, 1987). At least five independent transformants were selected. To produce *ProAlcA:MYB21*, the expression of MYB21 was driven by the ethanol-inducible *AlcA* promoter, and the resultant fragment was inserted into the pMLBart vector harboring a *Pro35S:AlcA* cassette (Roslan *et al.*, 2001). To produce *FLS1*-overexpressing (*FLS1OE*) *myb21*, the *FLS1* cDNA fragment was inserted into pCAMBIA1300 under the control of the 35S promoter. The overexpression of *FLS1* in the *myb21* mutant was able to rescue the infertility phenotype of *myb21* in 16 of 21 individual plants. These vectors were introduced into Arabidopsis plants by the *Agrobacterium tumefaciens*-mediated floral dip method (Clough *et al.*, 2010). The sequences of primers used in this investigation are listed in Supplementary Table S1.

### Plant treatments

For ethanol treatment, the *ProAlcA:MYB21* plants were grown in soil until flowering (5 weeks old). The plants were then sprayed with 1% ethanol or water (mock control). One hour later, plant materials were harvested and total RNA was extracted.

For the kaempferol and DPI treatments, 10 mM kaempferol (Sigma-Aldrich) and DPI (Sigma-Aldrich) in DMSO solution were used. Before floral stage 13 (flower in full bloom), the plants were sprayed with 2 μM kaempferol and 20 μM DPI five times at intervals of 2 days. The same concentration of DMSO was used as a mock treatment. The phenotypes of inflorescences and siliques were observed and analyzed.

### Expression analyses

For total RNA extraction, plant materials were homogenized in liquid nitrogen and mixed with Trizol reagent (Invitrogen) following the manufacturer’s instructions. First-strand cDNA was synthesized from 1 μg of total RNA using a reverse transcription system (TaKaRa). The fragments of interest were amplified by real-time PCR (qRT–PCR) using sequence-specific primers (Supplementary Table S1). qRT–PCR was conducted on an ABI7900HT Sequence Detection System (Applied Biosystems, Foster City, CA, USA) using GoTaq qPCR master mix (Promega) in accordance with the manufacturer’s instructions, and the gene expression level was normalized to that of *β-TUBULIN2* (*At5g62690*). The primers used in this study are listed in Supplementary Table S1.

### Dual-luciferase assay

Transient expression experiments and dual-luciferase assays were carried out as described previously (Liu *et al.*, 2008). The promoter region of *FLS1* was cloned into the pGreenII 0800-LUC vector. The *35S::REN* gene (*Renilla* luciferase) in the vector was used as an internal control. Equal concentrations and volumes of transformed *Agrobacterium* strains were mixed and co-infiltrated into *Nicotiana benthamiana* leaves by using a needleless syringe. After culturing for 3 days, the infiltrated leaves were harvested and the fluorescent values of firefly luciferase (LUC) and *Renilla* luciferase (REN) were analyzed using a Dual-Luciferase Reporter Assay System (Promega). The ratio of LUC to REN represented the activity of the *FLS1* promoter with or without the effect of the transcription factors MYB21, MYB24, and MYB57, as well as MYB12 and MYB99.

### Yeast one-hybrid assay

The PCR products of *MYB21* were amplified and purified, and full-length and truncated derivatives of the FLS1 promoter were inserted into a pHIS2.1 vector. MYB21-pGADT7-Rec (pGADT7-Rec plasmid digested by *Sma*I) was then co-transformed with bait vectors into yeast strain Y187 and plated on SD/-Leu/-Trp medium. Colonies were then transferred to SD/-Leu/-Trp/-His deficient medium with 25 mM 3-amino-1,2,4-triazole for 3 days.

### Chromatin immunoprecipitation

Approximately 1 g of seedlings of 10-day-old *Pro35S:MYB21-FLAG* and wild-type plants were harvested for chromatin immunoprecipitation (ChIP)–qRT assays. The ChIP experiments were performed using an EpiQuik^TM^ Plant ChIP Kit (EpiGentek, P-2014–48). According to the manufacturer’s instructions, the chromatin from the plant cells is extracted and sheared; the length of sheared DNA fragments is 200–500 bp. One-third of the crude chromatin extracts was saved for use as an input control, and the other two-thirds were treated with anti-FLAG antibody or (as a negative control) normal mouse IgG, separately. DNA was released from the antibody-captured protein–DNA complex and purified. Eluted DNA was further analyzed by qRT–PCR, with the *Actin8* promoter as a reference, as described (Yu *et al.*, 2010). The relative enrichment of each fragment was calculated first by normalizing the value for anti-FLAG against the value for the normal IgG control and then by comparing the result for *Pro35S:MYB21-FLAG* with that for the wild-type plants (Yu *et al.* 2015).

### Electrophoretic mobility shift assay

The full-length coding sequence of *MYB21* was cloned into the GST fusion vector (pGEX-4T-1) and then transformed into BL21 (DE3) *Escherichia coli*. The recombinant protein GST-MYB21 was purified using a Glutathione Sepharose 4 Fast Flow kit (GE Healthcare) according to the manufacturer’s protocol. The DNA fragments containing GARE motifs in the *FLS1* promoter were amplified using biotin-labeled primers (Supplementary Table S1) and purified using a PCR purification kit (Qiagen). The unlabeled wild-type (TTGTTA) and mutant (ATCTCC) probes were then tested for binding to GARE in competitive electrophoretic mobility shift assays (EMSAs). EMSA was performed using the Light Shift Chemiluminescent EMSA kit (Thermo Scientific) according to the manufacturer’s instructions. The migration of biotin-labeled probes was detected using an enhanced chemiluminescence substrate (Thermo Scientific) and the ChemDoc XRS system (Bio-Rad).

### *In situ* DPBA staining

For *in situ* visualization of flavonols, a method from Sheahan and Rechnitz (1992) was adapted. Inflorescences of plants were bleached with ethanol overnight at room temperature, and pollen grains were harvested from 10 flowers at late floral stage 12 to early stage 13 (just beginning to open) and stained with a freshly prepared aqueous solution of 0.25% (w/v) diphenylboric acid 2-aminoethylester (DPBA) and 0.00375% (v/v) Triton X-100 for at least 20 min. A Zeiss LSM LSM710 confocal laser scanning microscope was used to excite the pollen grains with 30% maximum laser power at 458 nm, and the fluorescence was collected at 475–504 nm for kaempferol and 577–619 nm for quercetin (Lewis *et al.*, 2011). Fluorescence was visualized on a Leica DM5500 B epifluorescence microscope (Leica, Wetzlar, Germany) with an excitation wavelength of 340–380 nm and a 425 nm long-pass splitter (Starcke *et al*., 2010).

### Flavonol extracts

Flavonol extracts were produced from ~100 mg of plant material in 2 ml reaction tubes by the addition of 0.5 ml of 80% methanol. Samples were frozen with liquid nitrogen and then homogenized. Homogenized samples were incubated for 15 min at 70 °C and centrifuged for 10 min at 15 000 g. The supernatants were transferred to new tubes and the extraction was repeated once. After the second extraction, the supernatants were combined and vacuum-dried at 35 °C.

### HPTLC with subsequent DPBA staining

A 1 μl volume of flavonol extract was spotted on to 10 cm ×10 cm silica-60 high-performance thin-layer chromatography (HPTLC) glass plates (Merck, Darmstadt, Germany). A system of ethyl acetate/formic acid/acetic acid/water (100:26:12:12, v/v/v/v) was chosen as the mobile phase for the chromatography (Starcke *et al*., 2010) in a closed glass tank. Separated flavonols were visualized by spraying the plates with a solution of 0.25% DPBA (w/v) in ethanol, followed by fast drying. The stained chromatograms were observed under UV light (Lewis *et al.*, 2011). The quantitative ultra-performance liquid chromatography-quadrupole-time of flight mass spectrometry (UPLC/Q-TOF MS) data of different flavonol derivatives are shown in Supplementary Table S2.

### Metabolic extraction determined by LC-MS

The profiling of the flavonols was implemented by an UPLC system (Agilent 1290) coupled to a Q-TOF mass spectrometer (Agilent 6545). The methods used were as described in previous studies (Yonekura-Sakakibara *et al.*, 2008; Stracke *et al.*, 2010) with modifications. Briefly, the dried pellets of flavonol extracts (prepared as described above) were dissolved in 300 μl of 80% methanol containing 3 μg of isovitexin as the internal standard. UPLC was performed on an Agilent Extend 300-C18 column (4.6 mm×150 mm×3.5 μm) with a flow rate of 0.8 ml min^–1^ at 30 °C. Compounds were separated by gradient elution with solvent A (0.2% acetic acid in water) and solvent B (0.2% acetic acid in acetonitrile) with the following elution profile: 0 min 98% A, 2% B; 1 min 98% A, 2% B; 2 min 85% A, 15% B; 15 min 78% A, 22% B; 17.5 min 50% A, 50% B; 19 min 100% B; 21.5 min 100% B; 22 min 98% A, 2% B.

The flavonol glycosides were determined by using a Q-TOF mass analyzer with a Dual AJS electrospray ionization (ESI) ion source. Full-scan mass spectra from 100 to 1000 *m*/*z* were used for the detection of [M + H]^+^ and the peak of fragment ions at one scan per second in a positive mode. Nitrogen gas was used as the sheath gas. The ESI-MS was performed at a gas temperature of 300 °C with a flow of 6 l min^–1^, nebulizer pressure 206 kPa, and spray voltage 3.5 kV.

The qualitative analyses were confirmed by comparison with reported data (Tohge *et al*., 2005, 2007; Routaboul *et al.*, 2006; Yonekura-Sakakibara *et al*., 2007, 2008), mainly including the retention time of the peak and the *m*/*z* values of flavonoid glycosides [M + H]^+^ with 10 V cone voltage and patterns of MS2 fragmentation under 20 V cone voltage (Supplementary Table S3).

### CM-H_2_DCFDA staining of pollen grains

Anthers were detached from 10 flowers during late flower stage 12 to early stage 13 (just beginning to open) and placed in a 2 ml conical tube. The pollen was then released from the anthers by vortexing (Muhlemann *et al.*, 2018; Luria *et al.*, 2019) and resuspended in pollen viability solution (PVS; 290 mM sucrose, 1.27 mM Ca(NO_3_)_2_, 0.16 mM boric acid, 1 mM KNO_3_) containing 5 μM 5-(and 6)-chroromethyl-2′,7′-dichlorodihydrofluorescein diacetate (CM-H_2_DCFDA) and 0.005% (v/v) Triton X-100. The PVS containing CM-H_2_DCFDA was then replaced with fresh PVS, and the stained pollen grains and stamen were placed on a microscope slide. Fluorescence was visualized on an inverted fluorescence microscope (ZEISS Axio Vert.A1) after excitation with a 488 nm laser. The quantification of viable pollen grains and stamens was performed using Fiji (Schindelin *et al.*, 2012).

### Statistical analysis

Differences were analyzed using Student’s *t*-test when comparing two variables and ANOVA with Fisher’s least significant difference (LSD) test when comparing three or more conditions. Values of *P*<0.05 were considered statistically significant. All analyses were performed using ORIGIN 8 software.

## Results

### MYB21 and its homologs positively regulate flavonol biosynthesis

**Fig. 1.**
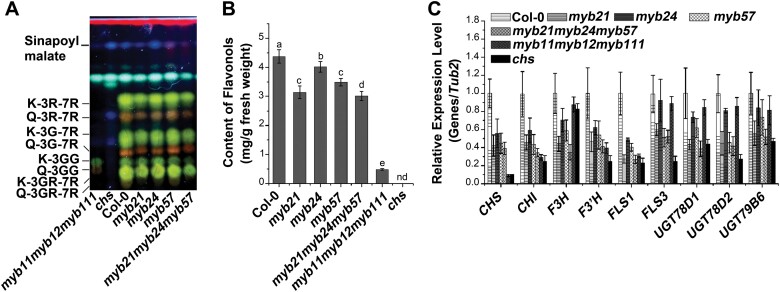
MYB mutations lead to reduced flavonol accumulation and flavonol synthase gene expression. (A) Flavonol glycoside accumulation patterns of the inflorescences of wild-type (Col-0) plants and *myb21*, *myb24*, *myb57*, *myb21myb24myb57*, *myb11myb12myb111*, and *chs* mutants. The flavonol accumulation of both *myb11myb12myb111* and *chs* mutants is included as a reference. Methanolic extracts were separated by HPTLC followed by DPBA staining and UV illumination. (B) Quantification of flavonols in the inflorescences of wild-type plants and *myb21*, *myb24*, *myb57*, *myb21myb24myb57*, *myb11myb12myb111*, and *chs* mutants. The flavonol accumulation of both *myb11myb12myb111* and *chs* mutants is included as a reference. nd, not detected. Error bars indicate the SD of three biological replicates. Different letters above the bars indicate significant differences in flavonol content between the wild type and other genotypes (*P*<0.05; ANOVA with Fisher’s LSD test). (C) Gene expression of flavonol biosynthesis genes in inflorescences of the wild type and the *myb21*, *myb24*, *myb57*, and *myb21myb24myb57* mutants. The related gene expression of both *myb11myb12myb111* and *chs* mutants is included as a reference. Transcripts were analyzed by qRT–PCR and *β-TUBULIN2* was used as the internal standard. Error bars indicate the SD of three biological replicates.

To explore whether the Arabidopsis stamen-enriched gene *MYB21* and its homologs (*MYB24* and *MYB57*) participate in flavonol biosynthesis, we quantified the flavonol content of inflorescences in the *myb* single mutants *myb21*, *myb24*, and *myb57* and in the triple mutant *myb21myb24myb57*. Two flavonoid-deficient mutants, *myb11myb12myb111* (lacking flavonols, except in the pollen grains) and *chs* (lacking all flavonoids), were used as positive controls. Analysis by HPTLC with subsequent DPBA staining showed that the inflorescences of the three *myb* single mutants accumulated less flavonol than the wild type, and the reduction in *myb21myb24myb57* was more drastic (Fig. 1A). These results were consistent with the results of LC-MS analysis (Fig. 1B).

We then investigated whether the transcription of flavonol biosynthesis genes was down-regulated in these *myb* mutants. Among the enzymes of flavonol biosynthesis, FLS is responsible for flavonol production, and *A. thaliana At5g08640* was previously characterized to encode a functional FLS, designated *FLS1*, whose transcripts were found to be enriched in developing inflorescences, floral buds, flowers, and siliques (Owens *et al.*, 2008). The expression of flavonol biosynthesis genes in Arabidopsis inflorescences was examined by qRT–PCR, which showed that the transcript levels of *CHS* (*AT5G13930*), *CHI* (*AT3G55120*), *F3H* (*AT3G51240*), *F3′H* (*AT5G07990*), *FLS1*, *FLS3* (*AT5G63590*), *UGT78D1* (*AT1G30530*), *UGT78D2* (*AT5G17050*), and *UGT79B6* (*AT5G54010*) were lower in the *myb21*, *myb24*, and *myb57* mutants, and down-regulated to a greater extent in the *myb21myb24myb57* triple mutant (Fig. 1C).

**Fig. 2.**
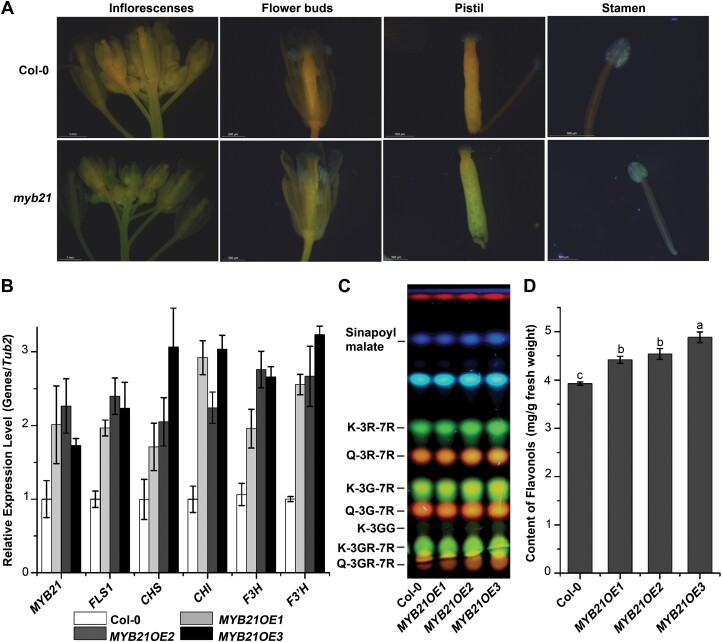
MYB21 and its homologs mediate flavonol biosynthesis through the regulation of flavonol synthase gene expression. (A) *In situ* flavonol staining of wild-type (Col-0) and *myb21* inflorescences. Flavonols in ethanol-bleached inflorescences were stained with DPBA to saturation and imaged by epifluorescence microscopy. Bars=1 mm (for inflorescences) or 500 μm (for buds, pistils, and stamens). (B) Gene expression of *FLS1*, *MYB21*, *CHS*, *CHI*, *F3H*, and *F3′H* in inflorescences of wild-type (Col-0) and *Pro35S:MYB21-FLAG* (*MYB21OE1–3*) plants. Transcripts were analyzed by qRT–PCR. Error bars indicate the SD of three biological replicates. (C) Flavonol glycoside accumulation patterns of the inflorescences of wild-type and *Pro35S:MYB21-FLAG* (*MYB21OE1–3*) plants. Methanolic extracts were separated by HPTLC followed by DPBA staining and UV illumination. (D) Quantification of flavonols in the inflorescences of wild-type and *Pro35S:MYB21-FLAG* (*MYB21OE1–3*) plants. Error bars indicate the SD of three biological replicates. Different letters above the bars indicate significant differences in flavonol content between the wild type and other genotypes (*P*<0.05; ANOVA with Fisher’s LSD test).

Redundant function and similar behavior have been reported among MYB21, MYB24, and MYB57 (Song *et al.*, 2011; Huang *et al.*, 2020). Given that the mutation of MYB21 alone causes defects in stamen development, leading to reduced fertility (Cheng *et al.*, 2009; Song *et al.*, 2011), we then focused on the role of MYB21 in *FLS1* expression, as a representative flavonol biosynthesis gene. After staining with DPBA, the developing flowers of wild-type plants exhibited yellow fluorescence, indicating the accumulation of flavonols. By contrast, in parts of *myb21* mutant flowers, including petals, pistils, filaments, and pollen, the flavonol accumulation was reduced (Fig. 2A). We found that the expression levels of *FLS1* were higher in *MYB21OE* transgenic plants (Fig. 2B). In addition, the transcript levels of *CHS*, *CHI*, *F3H*, and *F3′H* were up-regulated in the inflorescences of *MYB21OE* plants, indicating that *MYB21* might control the whole flavonol biosynthetic pathway (Fig. 2B). Consistently, the *MYB21OE* plants accumulated more flavonols than wild-type plants (Fig. 2C, D).

### MYB21 directly targets the *FLS1* promoter

**Fig. 3.**
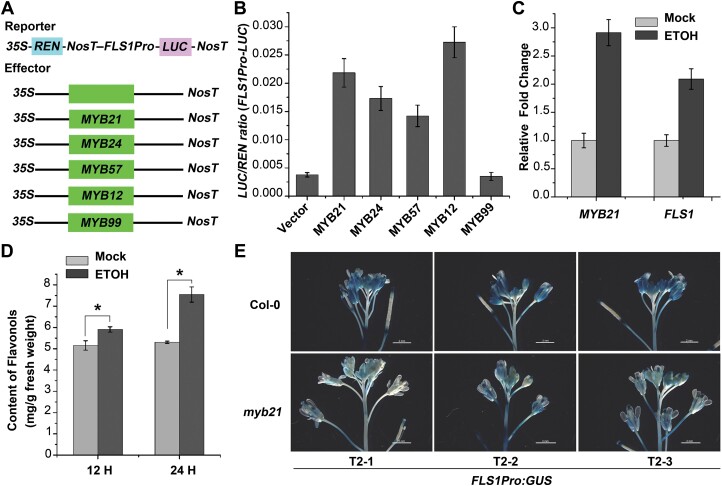
MYB21 directly regulates *FLS1* gene expression and final products by increasing promoter activity. (A) Schematic of the structures of effector (*35S:MYB21*, *35S:MYB24*, *35S:MYB57*, *35S:MYB12*, and *35S:MYB99*) and reporter (*FLS1Pro:LUC*) constructions for the dual-luciferase transient expression assay. The *35S:REN* gene (*Renilla* luciferase) in the pGreenII 0800-LUC vector was used as an internal control. (B) Relative LUC/REN ratio from the transient expression assays. The ratio of firefly luciferase (LUC) to REN represents the activity of the *FLS1* promoter in the absence or presence of MYB21 and its homologs. MYB12 was included as a positive control. Error bars indicate the SD of three biological replicates. (C) Expression of *MYB21* and *FLS1* in *ProAlcA:MYB21* inflorescences. The plants were sprayed with 1% ethanol, and the total RNA was isolated for analysis 1 h later. Error bars indicate the SD of three biological replicates. (D) Quantification of flavonols in the inflorescences of *ProAlcA:MYB21.* The plants were sprayed with 1% ethanol, and the flavonols were isolated after 12 h and 24 h of treatment. Error bars indicate the SD of three biological replicates. *P<0.05 (Student’s *t*-test). (E) GUS staining of inflorescences of *ProFLS1:GUS* in the wild-type (Col-0) and the *myb21* background. *ProFLS1:GUS T2-1/2/3* represents T_2_ generations of three individual transgenic lines, whereas *ProFLS1:GUS/myb21* plants were produced via crossing a single *ProFLS1:GUS* line with *myb21*. The florescence samples were incubated in GUS reaction mixture at 37 °C for 4 h. Bars=2 mm.

To determine how MYB21 and its homologs regulate the expression of *FLS1*, we performed a dual-luciferase assay by generating an *FLS1Pro-LUC* reporter. Here, we used MYB21, MYB24, and MYB57, as well as two other MYB transcription factors involved in flavonol biosynthesis (MYB12 and MYB99), under the control of the CaMV 35S promoter as effectors (Fig. 3A). The results showed that MYB21, MYB24, and MYB57 were functional in activating the *FLS1* promoter, and among these the activity of MYB21 was relatively higher (Fig. 3B). We also found that MYB12, but not MYB99, could promote the transcription of *FLS1* (Fig. 3B), which is consistent with a previous report (Mehrtens *et al.* 2005). We then used an alcohol-responsive promoter to generate *MYB21* transient-induction plants (*ProAlcA:MYB21*). A clear (nearly 2-fold) increase in the abundance of *FLS1* transcripts was observed 1 h after the application of 1% ethanol to flowers of the 5-week-old *ProAlcA:MYB21* plants (Fig. 3C). We found that the concentration of flavonols also increased after prolonged ethanol treatment (Fig. 3D). To further examine the regulatory role of MYB21 in *FLS1* expression, a *ProFLS1:GUS* plant was crossed with the *myb21* mutant. In the wild-type background, *ProFLS1:GUS* exhibited strong GUS activity in inflorescences, a pattern consistent with a previous report (Owens *et al.*, 2008). In the *myb21* background, however, the GUS staining in inflorescences was much fainter (Fig. 3E), which suggested that MYB21 regulates *FLS1* expression by acting on its promoter.

**Fig. 4.**
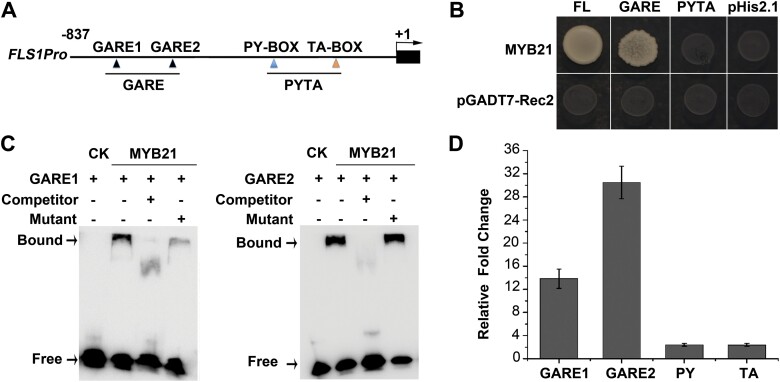
MYB21 recognizes and binds to the GARE elements of the *FLS1* promoter. (A) Schematic diagrams of the *FLS1* promoter constructs in yeast one-hybrid assays. Black triangles indicate GARE *cis*-elements (TTGTTA), and blue and orange triangles indicate the PY-BOX (TTTTTTCC) and TA-BOX (TATCCA), respectively. (B) Yeast one-hybrid assays of the interactions between MYB21 and *FLS1* promoter fragments. The empty vectors of pGADT7-Rec were used as a negative control. (C) EMSA indicating that MYB21 binds to the GARE motifs. Recombinant GST-MYB21 was purified from *E. coli*. The promoter fragment containing the two GARE motifs of the *FLS1* promoter was labeled with biotin. A competition assay for the protein–DNA binding was performed using 100× unlabeled wild-type (Competitor) and mutant probes. (D) ChIP enrichment of *FLS1* promoter regions bound by MYB21-FLAG. Ten-day-old *Pro35S:MYB21-FLAG* and wild-type (Col-0) seedlings were used; DNA fragments were analyzed by quantitative PCR, with the *Actin8* promoter as a reference. Enrichments in *Pro35S:MYB21-FLAG* were compared with wild-type seedlings. Error bars indicate the SD of three PCR repeats of four separate samples.

Since MYB21 is repressed by DELLA proteins and acts downstream of the GA pathway (Cheng *et al.*, 2009), we investigated whether it functions as GAMYB and directly binds to the GAREs in the *FLS1* promoter. Sequence analysis by PLACE (http://www.dna.affrc.go.jp/htdocs/PLACE/) revealed that the *FLS1* promoter (–837 bp) contains two GARE *cis*-elements (Fig. 4A), which could be recognized by GAMYB (Gubler *et al*., 1995, 1999). In a yeast one-hybrid assay, the full-length and truncated derivatives of the *FLS1* promoter were used as bait. We found that MYB21 bound to the full-length and GARE *cis*-elements of the *FLS1* promoter (Fig. 4B). We then performed EMSA using a recombinant GST-MYB21 protein produced in bacterial cells. We found that GST-MYB21 was able to bind to both GARE1 and GARE2 of the *FLS1* promoter. The specificity of this binding was further confirmed in a competition assay with excess amounts of unlabeled wild-type or mutant probes (Fig. 4C). To further demonstrate whether MYB21 could bind to the *FLS1* promoter *in vivo*, we performed a ChIP assay using a transgenic line that expressed a fusion of MYB21-FLAG under the control of the 35S promoter. Chromatin extracted from 10-day-old seedlings was immunoprecipitated with an antibody against FLAG. The presence of *FLS1* promoter sequences was analyzed by qPCR (Fig. 4D). Among the four regions we analyzed, two (GARE1 and GARE2) containing the GARE boxes were amplified in *35S:MYB21-FLAG* samples after pulldown with anti-FLAG antibodies. This suggests that MYB21 is able to bind to the promoter region of *FLS1*. Together, these results demonstrate that MYB21 promotes flavonol biosynthesis mainly through positive regulation of *FLS1* at the transcriptional level via the GARE domains in its promoter.

### MYB21-regulated flavonol accumulation is essential for stamen development

**Fig. 5.**
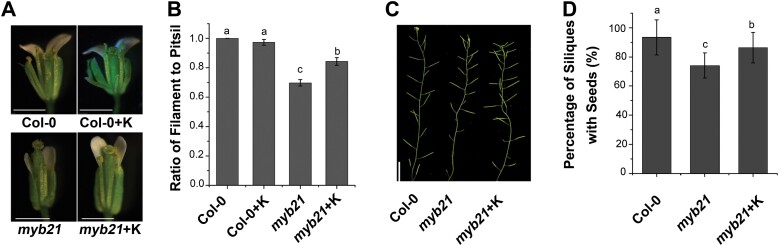
Treatment with kaempferol partially rescues stamen filament growth and fertility in the *myb21* mutant. (A) Phenotype of wild-type (Col-0) and *myb21* flowers with or without kaempferol (K) treatment. Bar=1 mm. (B) Ratio of filament length to pistil length in wild-type and *myb21* plants before and after kaempferol treatment. Error bars indicate the SD of three biological replicates. Different letters above the bars indicate significant differences between groups (*P*<0.05; ANOVA with Fisher’s LSD test). (C) Main shoot bearing siliques of wild-type and *myb21* plants with and without kaempferol treatment. (D) Percentage of siliques with seeds in wild-type and *myb21* plants with and without kaempferol treatment. Error bars indicate the SD of three biological replicates. Different letters above the bars indicate significant differences between groups (*P*<0.05; ANOVA with Fisher’s LSD test).

**Fig. 6.**
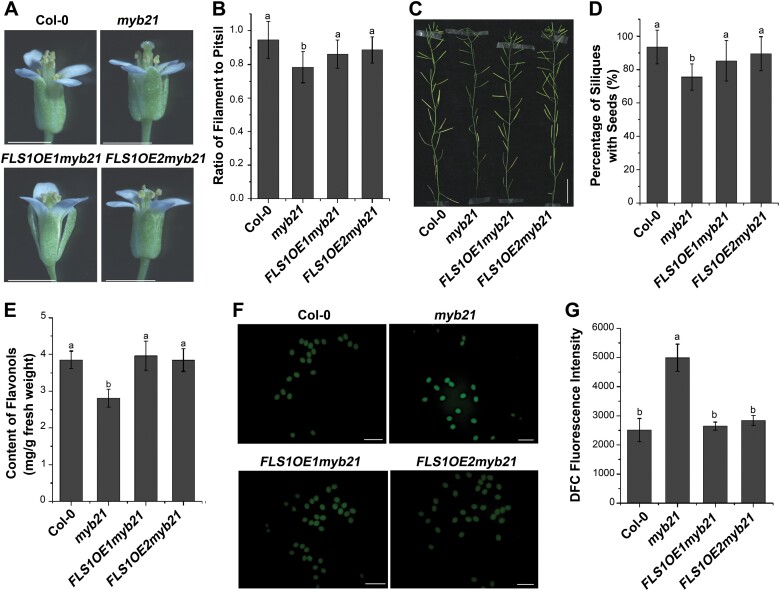
Overexpression of *Pro*35S:*FLS1* partially complements the phenotype of the *myb21* mutant. (A) Phenotype of wild-type (Col-0), *myb21*, *FLS1OE1myb21*, and *FLS1OE2myb21* flowers. Bar=1 mm. (B) Ratio of filament length to pistil length in wild-type, *myb21*, *FLS1OE1myb21*, and *FLS1OE2myb21* plants. Error bars indicate the SD of three biological replicates. Different letters above the bars indicate significant differences between groups (*P*<0.05; ANOVA with Fisher’s LSD test). (C) Main shoot bearing siliques of wild-type, *myb21*, *FLS1OE1myb21*, and *FLS1OE2myb21* plants. Bar=2 cm. (D) Percentage of siliques with seeds in wild-type, *myb21*, *FLS1OE1myb21*, and *FLS1OE2myb21* plants. Error bars indicate the SD of three biological replicates. Different letters above the bars indicate significant differences between groups (*P*<0.05; ANOVA with Fisher’s LSD test).(E) Quantification of flavonols in the inflorescences of wild-type, *myb21*, *FLS1OE1myb21*, and *FLS1OE2myb21* plants. Error bars indicate the SD of three biological replicates. Different letters above the bars indicate significant differences between groups (*P*<0.05; ANOVA with Fisher’s LSD test). (F) Images of pollen grains of wild-type, *myb21*, *FLS1OE1myb21*, and *FLS1OE2myb21* plants, stained with CM-H_2_DCFDA. Bar=50 μm. (G) Quantification of DCF fluorescence in pollen grains of wild-type plants and flavonoid biosynthesis mutants. Error bars indicate the SD of three biological replicates. Different letters above the bars indicate significant differences between groups (*P*<0.05; ANOVA with Fisher’s LSD test).

The *myb21* mutants have defects in filament elongation and pollen maturation, leading to stamen infertility (Song *et al.*, 2011; Qi *et al.*, 2015). Lack of flavonol biosynthesis has been reported to influence the germination of petunia pollen grains, which could be rescued by the addition of kaempferol, the main product of floral flavonols (Mo *et al.*, 1992). The regulation of the *FLS1* gene by MYB21 further encouraged us to investigate the effect of flavonol biosynthesis on stamen development. We therefore treated *myb21* flowers with 2 μM kaempferol before floral stage 13. The stamen development of *myb21* was distinguishable before and after kaempferol treatment (Fig. 5A). In *myb21*, the ratio of filament to pistil length was ~0.70 on average, but increased to ~0.84 after kaempferol application (Fig. 5B). We also evaluated the effect of kaempferol on seed production in the *myb21* mutant. After treatment with kaempferol, a higher proportion of the siliques in the *myb21* mutant contained seeds (Fig. 5C, D). To further confirm the contribution of flavonols to stamen development in the *myb21* mutant, we over-expressed *FLS1* driven by the 35S promoter in the *myb21* mutant. As expected, both transgenic plants (*FLS1OE1myb21* and *FLS1OE2myb21*) showed rescue of the defects in stamen development of *myb21* (Fig. 6A, B). Correspondingly, the seed production of the *myb21* mutant was increased to wild-type levels when *FLS1* was overexpressed in the transgenic plants (Fig. 6C, D).

### Excess ROS accumulation is involved in the stamen defects of the *myb21* mutant

**Fig. 7.**
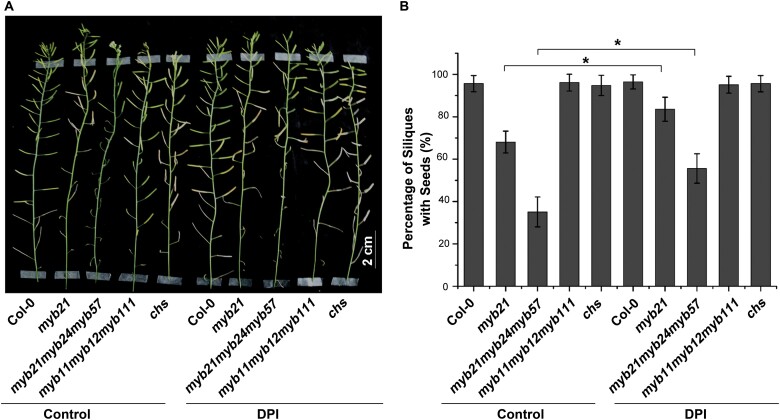
Treatment with DPI partially rescues stamen filament growth and fertility in the *myb21* and *myb21myb24myb57* mutants. (A) Main shoot bearing siliques of wild-type (Col-0), *myb21*, *myb21myb24myb57*, *myb11myb12myb111*, and *chs* plants with and without DPI treatment. (B) Percentage of siliques with seeds in wild-type, *myb21*, *myb21myb24myb57*, *myb11myb12myb111*, and *chs* plants with and without DPI treatment. Error bars indicate the SD of three biological replicates. *P<0.05 (Student’s *t*-test).

Although further research is needed to reveal how flavonol synthesis mediates stamen development, it has been hypothesized that flavonol accumulation contributes to plant fertility, probably due to involvement in the maintenance of ROS homeostasis (Potocký *et al.*, 2007; Duan *et al.*, 2014; Muhlemann *et al.*, 2018). As shown in Fig 6E, the concentration of flavonols in both *FLS1OE1myb21* and *FLS1OE2myb21* was higher than that in *myb21*, and was comparable to that in the wild type. To visualize and quantify total ROS, we treated the inflorescences with CM-H_2_DCFDA. The dichlorodihydrofluorescein (DCF) fluorescence intensity (a measure of ROS content) of pollen grains was higher in the *myb21* mutant than in the wild type (Fig. 6F, G). Importantly, DCF fluorescence intensity was reduced to wild-type levels in the *myb21* mutant when *FLS1* was overexpressed (i.e. in the transgenic plants *FLS1OE1myb21* and *FLS1OE2myb21*). To further verify the effect of ROS accumulation on stamen development in *myb* mutants, we treated *myb21* and *myb21myb24myb57* flowers with 20 μM DPI (a ROS inhibitor) before floral stage 13 (Huang *et al.*, 2019). After treatment with DPI, the siliques of the *myb21* and *myb21myb24myb57* mutants contained more seeds (Fig. 7). Although the cause of the excess ROS in *myb21* and *myb21myb24myb57*, but not in *myb11myb12myb111* or *chs*, needs further study, these data suggest that flavonols could prevent ROS from reaching damaging concentrations and restore impaired fertility.

### Pollen-specific flavonol accumulation contributes to male fertility by ROS scavenging in Arabidopsis

**Fig. 8.**
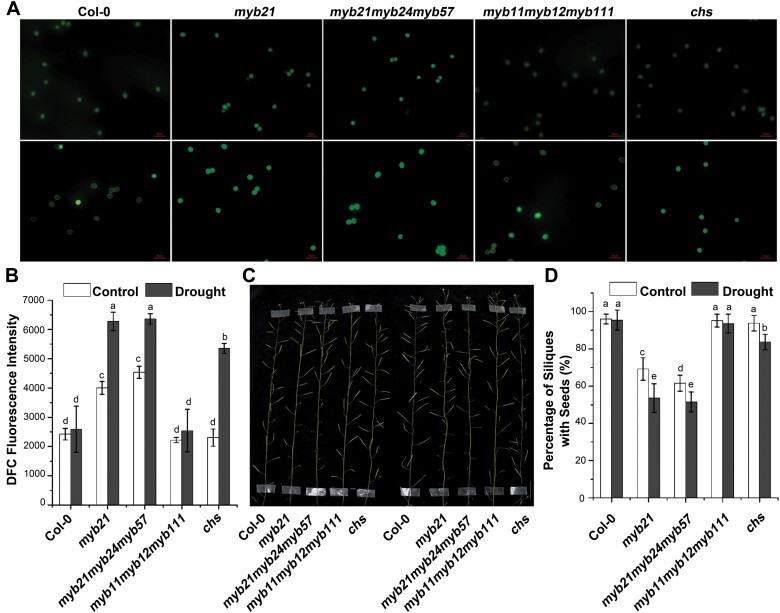
Pollen-specific flavonol accumulation contributes to male fertility by ROS scavenging in Arabidopsis. (A) Images of CM-H_2_DCFDA-stained pollen grains of wild-type (Col-0), *myb21*, *myb21myb24myb57*, *myb11myb12myb111*, and *chs* with and without drought treatment. Bars=50 μm. (B) Quantification of DCF fluorescence in pollen grains of the wild type and the flavonoid biosynthesis mutants. Different letters above the bars indicate significant differences between groups (*P*<0.05; ANOVA with Fisher’s LSD test). (C) Main shoot bearing siliques of wild-type, *myb21, myb21myb24myb57*, *myb11myb12myb111*, and *chs* plants with and without drought treatment. (D) Percentage of siliques with seeds in wild-type, *myb21*, *myb21myb24myb57*, *myb11myb12myb111*, and *chs* plants with and without drought treatment. Error bars indicate the SD of three biological replicates. Different letters above the bars indicate significant differences between groups (*P*<0.05; ANOVA with Fisher’s LSD test).

Plant reproductive organs are known to be highly sensitive to drought, which might result from the accumulation of excess ROS (Su *et al.*, 2013). To further support a plausible hypothesis that flavonols mediate stamen development by regulating ROS homeostasis in Arabidopsis, we treated plants including wild type, *myb21*, *myb21myb24myb57*, *myb11myb12myb111*, and *chs* with drought, as described by Su *et al.*, 2013. The results showed that drought increased the DCF fluorescence of pollen grains 1.5-fold in *myb21*, 1.4-fold in *myb21myb24myb57*, and 2-fold in *chs*. However, the pollen grains in both the wild type and the *myb11myb12myb111* mutant maintained relatively stable ROS with and without drought (Fig. 8A, B). To investigate whether the higher ROS levels in *myb21*, *myb21myb24myb57*, and *chs* than those in *myb11myb12myb111* and *chs* resulted in impaired fertility, we estimated the percentage of siliques with seeds in wild type, *myb21*, *myb21myb24myb57*, *myb11myb12myb111*, and *chs* plants. As expected, *myb21*, *myb21myb24myb57*, and *chs* showed a reduction of nearly 10% of siliques with seeds in response to drought, whereas drought did not affect fertility in the wild type or in *myb11myb12myb111* (Fig. 8C, D). These results suggest that the enhanced sensitivity of flavonol biosynthesis mutants to drought -induced infertility was due to elevated ROS.

Together, our data demonstrate that the transcription factor MYB21 and its homologs (MYB24 and MYB57) regulate the expression of *FLS1* at the transcriptional level. Furthermore, we propose that MYB21-mediated flavonol accumulation is involved in stamen development and seed production by ROS scavenging in Arabidopsis, which provides a new insight into the biological functions of flavonols.

## Discussion

Recently, members of subgroup 7 of the R2R3-MYB transcription factors, namely MYB11, MYB12, and MYB111, were identified as flavonol-specific regulators of flavonoid biosynthesis in Arabidopsis (Mehrtens *et al.*, 2005; Stracke *et al*., 2007, 2010). However, the expression pattern of *FLS1* does not follow those of the subgroup 7 *MYB*s (Stracke *et al.*, 2010). The flower-enriched pattern of *FLS1* expression implies the involvement of other, tissue-specific, transcription factors. MYB21 and its homologs, which belong to subgroup 19 of the R2R3-MYB family, are enriched in the stamen and are involved in the regulation of stamen development (Cheng *et al.*, 2009). However, the downstream pathways of these MYBs need further investigation in Arabidopsis. In this report, we identified that MYB21 and its homologs regulate the gene expression of FLS1, the key enzyme in the catalysis of dihydroflavonols into flavonols (Wisman 1998; Owens *et al.*, 2008). In *myb21* mutants, flavonol accumulation in inflorescences was reduced, and the reduction was even more pronounced in *myb21myb24myb57* mutants (Fig. 1A, B). Considering the characteristic stamen phenotype of *myb21*, the enrichment of *MYB21* transcripts in sepals, petals, stamen, and stigma, but not in roots or leaves (Supplementary Fig. S2), further convinced us to focus on MYB21-mediated flavonol biosynthesis in inflorescence. The flavonol content of *myb21* suggested a relatively minor regulatory role of MYB21 on flavonol biosynthesis (Fig. 1). However, we found that *MYB24* transcripts were enriched in the pistils and stamen of *myb21*, reaching expression levels that paralleled those of the wild type (Supplementary Fig. S3). As we have pointed out, MYB24 is homologous to MYB21, and mediated flavonol accumulation in Arabidopsis flowers. In addition, *MYB21* transcripts were still detectable in the *myb21* mutant, although at a much lower level than that in the wild type. Given that *myb21* is likely a leaky allele, an additional possibility is that the residual function of MYB21 could partially promote *FLS1* expression as well as flavonol accumulation. Furthermore, the *myb21* mutants still contained some flavonols in pollen, implying that the SG19 Myb regulators might not be the exclusive regulators involved in pollen flavonol biosynthesis.

Previously, MYB21 and its homologs were characterized as GAMYB transcription factors and were thought to be involved in GA responses. The development of floral organs is impaired in GA-deficient plants, which indicates that GA influences the biosynthesis of flavonols (Koorneef *et al.*, 1980; Cheng *et al.*, 2009). Previous microarray data support our hypothesis, since they showed that paclobutrazol, an inhibitor of GA biosynthesis, decreased the transcript levels of *FLS1* (Owens *et al.*, 2008). Providing further support, it was also reported that the DELLA proteins, which are repressors of the GA pathway, interact with MYB21 and MYB24 to regulate filament elongation in Arabidopsis (Huang *et al.*, 2020). Here, we present evidence that, following the mode of classic GAMYB, MYB21 can directly bind to the GAREs within the *FLS1* promoter and regulate the expression of this gene (Fig. 4). *UGT73B2*, another flavonol biosynthetic gene, has been proposed to be under the control of MYB21 (Kim *et al.*, 2006; Battat *et al.*, 2019). This gene contains GARE elements in its promoter region, which supports our finding. Although it has been reported that MYB99 could act in a regulatory triad with MYB21 and MYB24 to mediate phenylpropanoid biosynthesis (Battat *et al.*, 2019), the failure of MYB99 to activate the *FLS1* promoter (Fig. 3A, B) suggests that MYB21/MYB24/MYB57 and MYB99 have different specific direct target genes.

The GARE element also exists in the promoters of other genes encoding enzymes involved in the phenylpropanoid pathway, such as *CAD6* (*AT4G37970*), *F5H* (*AT4G36220*), and *CCoAoMT* (*AT4G34050*), which is responsible for lignin monolignol biosynthesis (Weng *et al.* 2010). The defect in lignified thickening leads to male sterility (Thévenin *et al*., 2011). We found that the expression of these three lignin biosynthetic genes was repressed in *myb* mutants, whereas it was induced in *MYB21OE* plants (Supplementary Fig. S4). HPTLC staining showed a consistent pattern of sinapoyl malate accumulation (blue spots; Figs 1A, 2C) and further suggested an effect of MYB21 on lignin deposition. In land plants, the phenylpropanoid pathway provides the precursor 4-coumaroyl CoA for the biosynthesis of lignins and flavonoids. The fine mediation of the branched phenylpropanoid biosynthetic metabolic flux is essential for plant growth and development, where when lignin biosynthesis is overactivated flavonoid biosynthesis is repressed (Besseau *et al.*, 2007; Owens *et al.*, 2008; Peng *et al.*, 2008, Stracke *et al.*, 2009; Gou *et al.*, 2011). In this case, the transgenic plants (*MYB21OE1–3*) overexpressing *MYB21* exhibited different induction effects on *FLS1* and *F5H* (Fig. 2BSupplementary Fig. S4B). Hence, it was reasonable that the highest expression of *FLS1* was detected in *MYB21OE3*, while the highest expression of lignin biosynthesis genes was detected in *MYB21OE1*. Furthermore, the excessive expression of *MYB21* leads to a reduction in fertility (Song *et al.*, 2011). Consistently, we also found that in *FLS1OEmyb21* transgenic plants with rescued fertility, the moderate expression level of *FLS1* was approximately twice that of *myb21* and almost equal to that of the wild-type (Supplementary Fig. S5). Additionally, we analyzed the expression of lignin biosynthetic genes in *chs* and *myb11myb12myb111.* We observed that the filament length of *chs* and *myb11my12myb111*, two flavonol biosynthesis mutants with normal fertility, resembled that of the wild type (Supplementary Fig. S6). The qRT–PCR results indicated that lignin production in *chs* and *myb11my12myb111* mutants was not affected (Supplementary Fig. S4A). All these results suggested that suitable contents and proportions of flavonols and of these other metabolites are important for stamen development.

The difference in lignin biosynthesis between *myb21myb24myb57* and *myb11myb12myb111* reminds us of the coordination between these MYBs in regulating flavonol biosynthesis. First, the qRT–PCR results showed that the expression level of *MYB11*, *MYB12*, and *MYB111* was not altered significantly in the *myb21* (or *myb21myb24myb57*) mutant, and neither was the level of *MYB21* transcripts in *myb11myb12myb111* (Supplementary Fig. S7A). It has been pointed out that flavonol accumulation is impaired in the pollen grains of *myb21* and *myb21myb24myb57*, but not in *myb11myb12myb111* (Supplementary Fig. S7B). Finally, we also noticed that the down-regulation of early biosynthesis enzyme genes, such as *CHS* and *CHI*, was more pronounced in *myb11myb12myb111* than in *myb21myb24myb57*, whereas the decrease of late biosynthesis enzyme gene expression was equivalent between *myb11myb12myb111* and *myb21myb24myb57* (Fig. 1C). Since all flavonols are ultimately derived from chalcone and subsequent flavanones, the limit of a precursor reasonably leads to a defect in flavonol production that is much more severe in *myb11myb12myb111* than that in *myb21myb24myb57* (Fig. 1A, B). The flavonol biosynthetic pathway, from CHS to FLS1, which leads to the formation of the flavonol aglycone, is common throughout the whole plant. In addition, MYB11-, MYB12-, and MYB111-independent synthesis of the pollen-specific metabolites Q-3GG and K-3GG further indicate that UGTs, which are responsible for the synthesis of flavonol glycosides, are under the control of an unknown regulator (Stracke *et al.*, 2010; Yonekura-Sakakibara *et al.* 2014). Interestingly, the transcripts of *UGT*s decreased more significantly in *myb21myb24myb57* than in *myb11myb12myb111* (Fig. 1C), implying a major regulatory effect of MYB21/MYB24/MYB57 on the expression of *UGT*s. All these observations hint that the regulatory action of MYB21/MYB24/MYB57 on flavonoid biosynthesis was at least partly independent of MYB11/MYB12/MYB111.

These flavonols are ubiquitous specialized metabolites of plants with varying degrees of hydroxylation, which possess antioxidant activity and thereby act as ROS scavengers (Winkel-Shirley *et al.*, 2001; Pourcel *et al.*, 2007; Hernández *et al.*, 2009). Consistently, mutants with decreased levels of flavonols contain elevated levels of ROS in root hairs and guard cells (Buer *et al.*, 2009; Watkins *et al.*, 2017). Additionally, ROS have been implicated in reproduction, with effects on stamen development (Potocký *et al.*, 2007; Kaya *et al.*, 2014; Xie *et al.*, 2014). Furthermore, several studies have made a link between reduced flavonol levels and impaired fertility in petunia, maize, tobacco, and tomato, but not in Arabidopsis (Mo *et al.*, 1992; Ylstra *et al.*, 1992; Pollak *et al.*, 1993; Burbulis *et al.*, 1996; Ylstra *et al.*, 1996). In maize and petunia, the exogenous application of flavonols could rescue the infertility of a *chs* mutant (Mo *et al.*, 1992; Taylor *et al.*, 1992; Pollak *et al.*, 1995). Consistently, the suppression of *FLS* expression reduced seed set in tobacco (Mahajan *et al.*, 2011). More recently, the tomato *are* mutant, which is defective in flavonol biosynthesis, was reported to exhibit impaired pollen grains and reduced seed yield (Muhlemann *et al.*, 2018). An *OsCHS1* T-DNA insertion mutant (*oschs1*) with no seed formation was characterized, and results indicated that flavonols were essential for male fertility in rice (Wang *et al.*, 2020). Here, we showed that the pollen grains of the *myb21* mutant accumulated less flavonols and had a higher abundance of ROS than pollen grains of the wild type. These findings suggested that flavonols act as ROS scavengers in reproductive tissues (Fig. 6). Treatment with DPI, which blocks ROS synthesis, rescued fertility defects in the *myb21* mutant (Fig. 7), consistent with the mediation of stamen development by flavonols through their ROS-scavenging properties. To uncover why *myb11myb12myb111* and *chs*, which have pronounced defects in flavonol biosynthesis (Fig. 1), exhibited normal stamen development, we treated the flavonol-biosynthesis mutants *myb21, myb21myb24myb57*, *myb11myb12myb111*, and *chs* with drought. The results showed that *myb21*, *myb21myb24myb57*, and *chs* exhibited defects in pollen-specific flavonol biosynthesis along with higher drought-induced ROS accumulation, which resulted in greater sensitivity of male fertility to drought (Fig. 8). Although the cause of the overaccumulation of ROS in *myb21* grown in normal conditions, but not in *chs* or *myb11myb12myb111*, is unclear, our work has enabled the development of a plausible and testable hypothesis that flavonols mediate stamen development by regulating ROS homeostasis in Arabidopsis. There are other hypotheses that flavonols regulate auxin concentration and transport, which is essential for reproductive development (Kuhn *et al.*, 2011; Lewis *et al.*, 2011; Zhao *et al.*, 2013; Cardarelli *et al.*, 2018; Tan *et al.*, 2019). In conclusion, our study reveals that the regulation of flavonol biosynthesis is orchestrated by MYB21 or its homologs, and contributes to stamen development and male sterility in Arabidopsis.

## Supplementary data

The following supplementary data are available at *JXB* online.

Fig. S1. Overexpression of *Pro*35S:*MYB21-FLAG* complements the phenotype of the *myb21* mutant.

Fig. S2. Transcription of *MYB21* is enriched in Arabidopsis flowers.

Fig. S3. Transcripts of *MYB21* and *MYB24* are detectable in the stamen and pistils of *myb21.*

Fig. S4. MYB21 is involved in the regulation of lignin biosynthesis genes.

Fig. S5. Gene expression of *FLS1* in inflorescences of wild type (Col-0), *myb21*, *FLS1OE1myb21*, and *FLS1OE2myb21*.

Fig. S6. *myb11myb12myb111* and *chs* plants show normal stamen development.

Fig. S7. MYB21 and MYB11/MYB12/MYB111 probably mediate the biosynthesis of phenylpropanoid metabolites in their own distinct way.

Table S1. Oligonucleotide primer sequences.

Table S2. Quantitative UPLC/Q-TOF MS data of different flavonol derivatives.

Table S3. Flavonol profiles in methanol–water extracts.

erab156_suppl_Supplementary_Figures_S1-S7_Table_S1-S3

## Data Availability

The data supporting the findings of this study are available from the corresponding author, Gaojie Hong, upon request.
