# Supplementary material for: Involvement of the R2R3-MYB transcription factor MYB21 and its homologs in regulating flavonol accumulation in Arabidopsis stamen
Source: J Exp Bot. 2021 Apr 8;72(12):4319–32. doi: 10.1093/jxb/erab156 (PMC8163065; doi:10.1093/jxb/erab156)
Supplement: erab156_suppl_Supplementary_Figures_S1-S7_Table_S1-S3 [file erab156_suppl_supplementary_figures_s1-s7_table_s1-s3.pdf]

MYB21-Mediated Flavonol Accumulation Contributes to Stamen Development by ROS Scavenging in *Arabidopsis*

Xueying Zhang[1,#], Yuqing He[1,#], Linying Li[1],Gaojie Hong[1,*]

[1] State Key Laboratory for Managing Biotic and Chemical Threats to the Quality and Safety of Agro-products, Institute of Virology and Biotechnology, Zhejiang Academy of Agricultural Sciences, 198 Shiqiao Road, Hangzhou 310021, China

[#] These authors contributed equally to this work

* Corresponding author

Supplementary data

Fig. S1. Overexpression of *Pro*35S:*MYB21-FLAG* could complement the phenotype of *myb21* mutant. (A) Flowers of the wild-type (Col-0), *myb21* , *Pro35S:MYB21-FLAG/myb21* transgenic plants (*MYB21OE myb21* T2 1-3). (B) Main shoot bearing siliques of wild-type (Col-0), *myb21*, *Pro35S:MYB21-FLAG over-expressing in myb21* (*MYB21OE*/*myb21* T2 1-3).

Fig. S2. The transcription of MYB21 is enriched in *Arabidopsis* flowers. The gene expression of *MYB21* in root, leaves, sepals, stamen and stigma, respectively, were analyzed by qRT-PCR, and *β-TUBULIN2* was used as the internal standard. Error bars indicate SD of three biological replicates.

Fig. S3. The transcripts of *MYB21* and *MYB24* could be detectable in the stamen and pistils of *myb21*. The gene expression of *MYB21* and *MYB24* was analyzed by qRT-PCR, and *β-TUBULIN2* was used as the internal standard. Error bars indicate SD of three biological replicates. *Student's test, $P < 0.05$.

Fig. S4. MYB21 was involved in the regulation on lignin biosynthesis genes. (A) Expression of lignin biosynthesis genes in inflorescences of wild-type (Col-0), *myb21*, *myb24*, *myb57* and *myb21myb24myb57*, *myb11myb12myb111* and *chs* mutants. The transcripts were analyzed by qRT-PCR, and *β-TUBULIN2* was used as the internal standard. Error bars indicate SD of three biological replicates. (B) Expression of lignin biosynthesis genes in inflorescences of wild-type (Col-0) and *MYB21* over-expression plants (*MYB21OE1-3*). The transcripts were analyzed by qRT-PCR, and *β-TUBULIN2* was used as the internal standard. Error bars indicate SD of three biological replicates.

Fig. S5. Gene expression of *FLS1* in inflorescences of wild type (Col-0), *myb21*, *FLS1OE1myb21*and *FLS1OE2myb21*, respectively. Error bars indicate SD of three biological replicates.

Fig. S6. Both *myb11myb12myb111* and *chs* plants shows normal stamen development. (A) Phenotype of flowers. (B) Main shoot bearing siliques. (C) The ratio of filament length to pistil length. Error bars indicate SD of three biological replicates. Bars marked by different letters are significantly different, $P < 0.05$. (D) Percentage of silique with seeds. Error bars indicate SD of three biological replicates. Bars marked by different letters are significantly different, $P < 0.05$.

Fig. S7. MYB21 and MYB11/MYB12/MYB111 probably mediate the biosynthesis of phenylpropanoid metabolites in their own distinct way. (A) Gene expression of *MYB11*, *MYB12*, *MYB111* and *MYB21* in inflorescences of wild-type (Col-0), *myb21*, *myb21myb24myb57* and *myb11myb12myb111*, respectively. (B) *In situ* flavonol staining of wild-type (Col-0), *myb21*, *myb21myb24myb57* and *myb11myb12myb11* pollen grains, respectively. Flavonols in ethanol-bleached inflorescences were stained with diphenylboric acid 2-aminoethylester (DPBA) to saturation and imaged by inverted fluorescence microscope.

Table S1. Oligonucleotide primer sequences.

Table S2. The quantitative UPLC/Q-TOF MS data of different flavonol derivatives.

Table S3. The flavonol profiles in methanol-water extracts.

Supplementary Figures

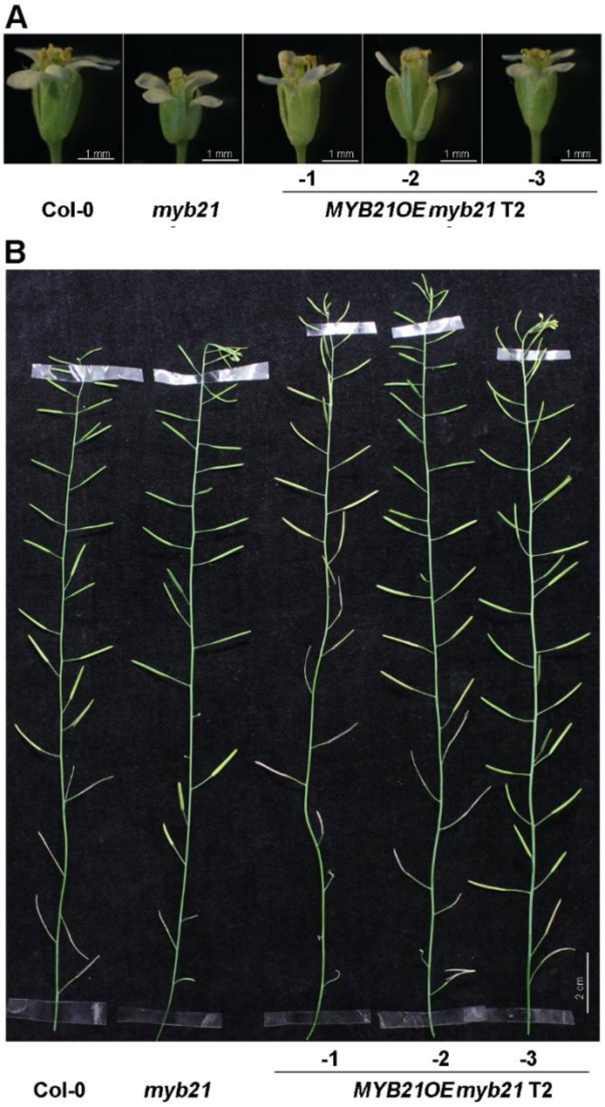

Fig. S1. Overexpression of *Pro*35S:*MYB21-FLAG* could complement the phenotype of *myb21* mutant. (A) Flowers of the wild-type (Col-0), *myb21* , *Pro35S:MYB21-FLAG/myb21* transgenic plants (*MYB21OE myb21* T2 1-3). (B) Main shoot bearing siliques of wild-type (Col-0), *myb21*, *Pro35S:MYB21-FLAG over-expressing in myb21* (*MYB21OE*/*myb21* T2 1-3).

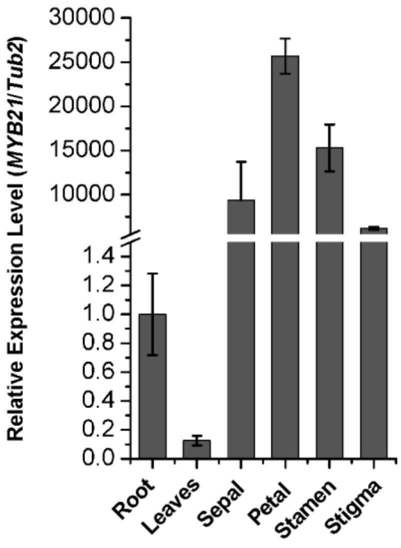

Fig. S2. The transcription of MYB21 is enriched in *Arabidopsis* flowers. The gene expression of *MYB21* in root, leaves, sepals, stamen and stigma, respectively, were analyzed by qRT-PCR, and *β-TUBULIN2* was used as the internal standard. Error bars indicate SD of three biological replicates.

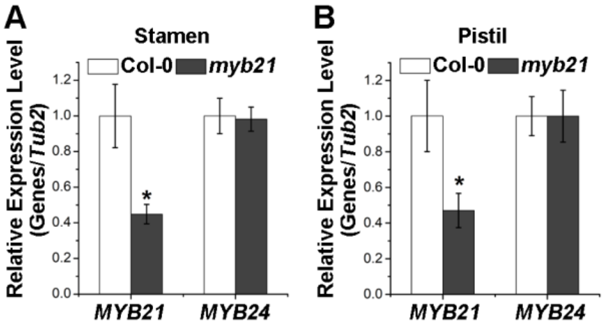

Fig. S3. The transcripts of *MYB21* and *MYB24* could be detectable in the stamen and pistils of *myb21*. The gene expression of *MYB21* and *MYB24* was analyzed by qRT-PCR, and *β-TUBULIN2* was used as the internal standard. Error bars indicate SD of three biological replicates. *Student's test, $P < 0.05$.

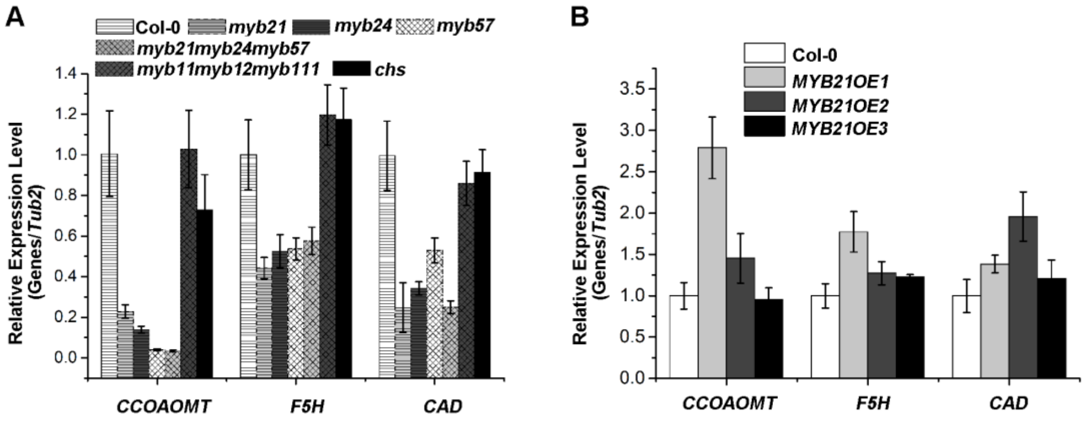

Fig. S4. MYB21 was involved in the regulation on lignin biosynthesis genes. (A) Expression of lignin biosynthesis genes in inflorescences of wild-type (Col-0), *myb21*, *myb24*, *myb57* and *myb21myb24myb57*, *myb11myb12myb111* and *chs* mutants. The transcripts were analyzed by qRT-PCR, and *β-TUBULIN2* was used as the internal standard. Error bars indicate SD of three biological replicates. (B) Expression of lignin biosynthesis genes in inflorescences of wild-type (Col-0) and *MYB21* over-expression plants (*MYB21OE1-3*). The transcripts were analyzed by qRT-PCR, and *β-TUBULIN2* was used as the internal standard. Error bars indicate SD of three biological replicates.

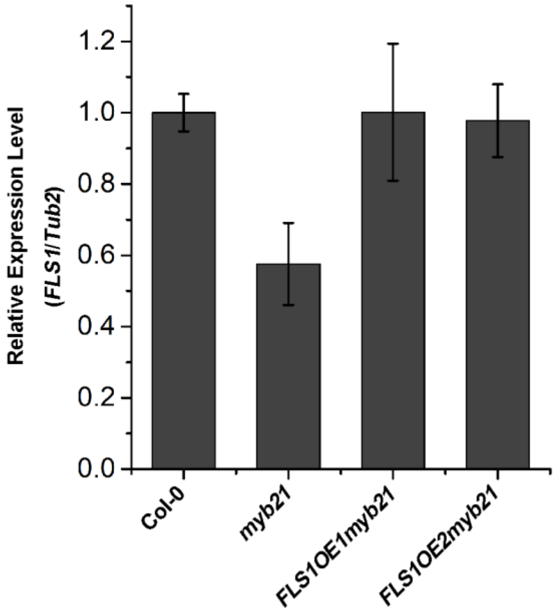

Fig. S5. Gene expression of *FLS1* in inflorescences of wild type (Col-0), *myb21*, *FLS1OE1myb21*and *FLS1OE2myb21*, respectively. Error bars indicate SD of three biological replicates.

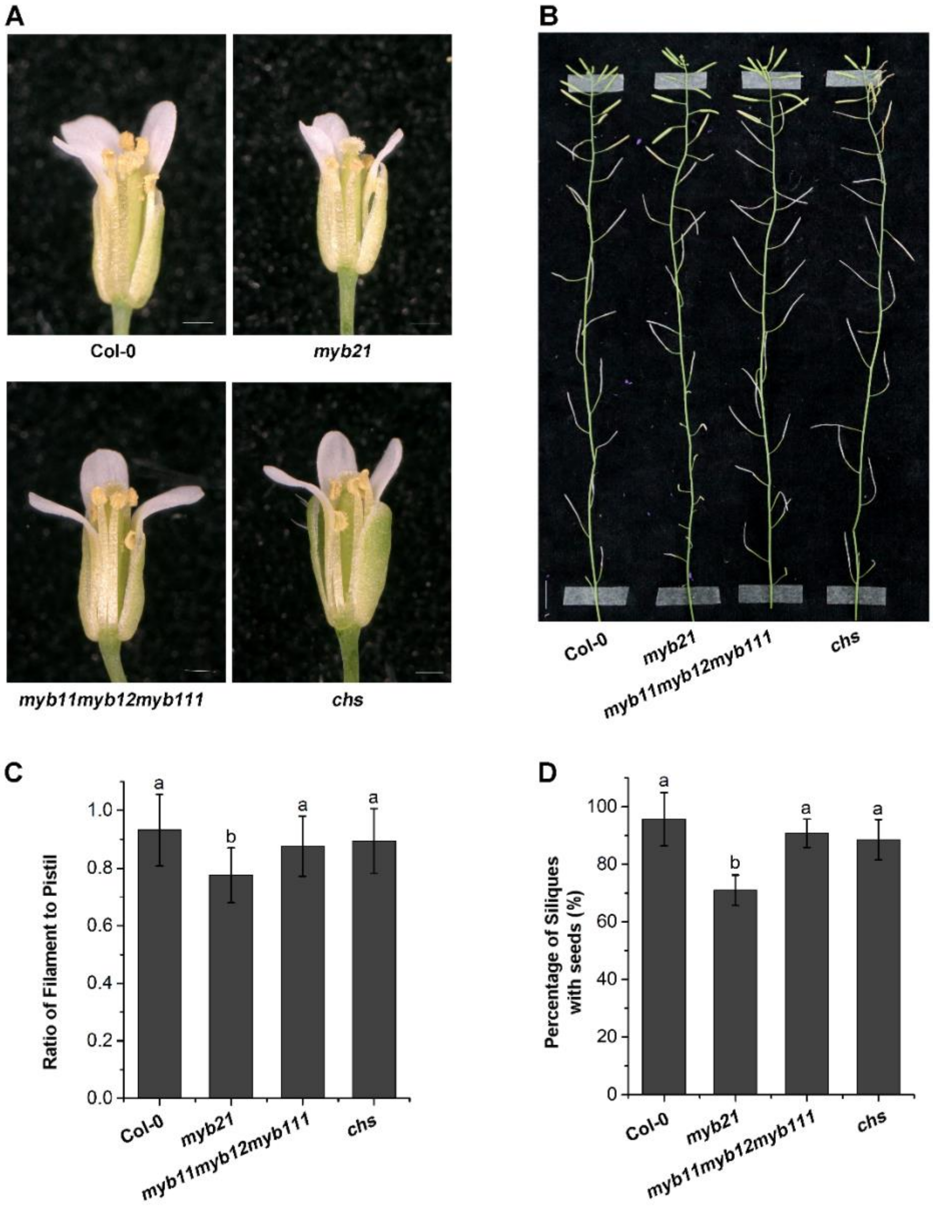

Fig. S6. Both *myb11myb12myb111* and *chs* plants shows normal stamen development. (A) Phenotype of flowers. (B) Main shoot bearing siliques. (C) The ratio of filament length to pistil length. Error bars indicate SD of three biological replicates. Bars marked by different letters are significantly different, P < 0.05. (D) Percentage of silique with seeds. Error bars indicate SD of three biological replicates. Bars marked by different letters are significantly different, P < 0.05.

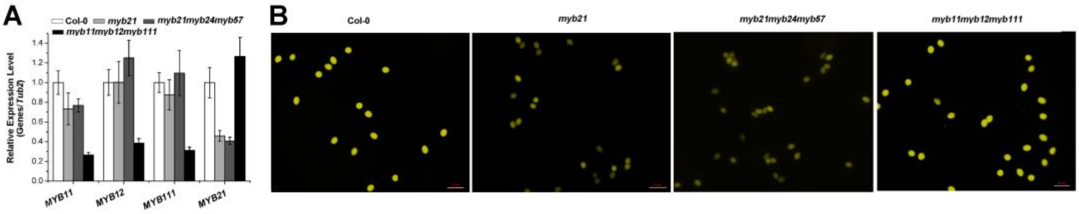

Fig. S7. MYB21 and MYB11/MYB12/MYB111 probably mediate the biosynthesis of phenylpropanoid metabolites in their own distinct way. (A) Gene expression of *MYB11*, *MYB12*, *MYB111* and *MYB21* in inflorescences of wild-type (Col-0), *myb21*, *myb21myb24myb57* and *myb11myb12myb111*, respectively. (B) *In situ* flavonol staining of wild-type (Col-0), *myb21*, *myb21myb24myb57* and *myb11myb12myb11* pollen grains, respectively. Flavonols in ethanol-bleached inflorescences were stained with diphenylboric acid 2-aminoethylester (DPBA) to saturation and imaged by inverted fluorescence microscope.

Table S1. **Primers used in this study**

Primer names	Primer sequence
Pro35S:MYB21-FLAG-F	ATGGAGAAAAGAGGAGGAGGAAG
Pro35S:MYB21-FLAG-R	ATTACCATTCAATAAATGCATTG
ProALcA:MYB21-F	GGGGTACCAATATGCGAATTAGCCTCTCACC
ProALcA:MYB21-R	ACGCGTCGACTATTTTTTTTGGTAGTTTGCGTTGCC
ProFLS-GUS-F	CCGCTCGAGAATATGCGAATTAGCCTCTCACC
ProFLS-GUS-R	CGGGATCCTATTTTTTTTGGTAGTTTGCGTTGCC
MYB21-GST-F	CGGGATCCATGGAGAAAAGAGGAGGAGGAAG
MYB21-GST-R	ACGCGTCGACTCAATTACCATTCAATAAATGCA
GARE1-EMSA-F	AGCCAGTGGCGATAAGCTCTCACCTTTTTTTTGTTATTTTATATCTTAAG
GARE1-EMSA-R	CTTAAGATATAAAATAACAAAAAAAAGGTGAGAGCTTATCGCCACTGGCT
GARE1mut-EMSA-F	AGCCAGTGGCGATAAGCTCTCACCTTTTTTATCTCCTTTTATATCTTAAG
GARE1mut-EMSA-R	CTTAAGATATAAAAGGAGATAAAAAAGGTGAGAGCTTATCGCCACTGGCT
GARE2-EMSA-F	AGCCAGTGGCGATAAGCTGGGCCTTGTCTTGTTATATGGGCCTTGAATC
GARE2-EMSA-R	GATTCAAGGCCCATATAACAAGACAAGGCCCAGCTTATCGCCACTGGCT
GARE2mut-EMSA-F	AGCCAGTGGCGATAAGCTGGGCCTTGTCATCTCCTATGGGCCTTGAATC
GARE2mut-EMSA-R	GATTCAAGGCCCATAGGAGATGACAAGGCCCAGCTTATCGCCACTGGCT
biotin-labeled probes	AGCCAGTGGCGATAAG
pHis2.1:FLSpro-F	CGGAATTCAATATGCGAATTAGCCTCTCACC
pHis2.1:FLSpro-R	GACTAGTTATTTTTTTTGGTAGTTTGCGTTGCC
pHis2.1:GARE-F	CGGAATTCGCCTCTCACCTTTTTTTTGTTA
pHis2.1:GARE-R	GACTAGTCAAGGCCCATATAACAAGACAAG
pHis2.1:PYTA-F	CGGAATTCAACATCACTTTTTTCCTTTTC

pHis2.1:PYTA-R	GACTAGTGAAATCTTGGCCGCTGGATAGAG
MYB21rec-F	GAATTCCACCCAAGCAGTGGTATCAACGCAGAGTGGATGGAGAAAAGAGGAGGAGGAAG
MYB21rec-R	ATCGATGCCCACCCTCTAGAGGCCGAGGCGGCCGACTCAATTACCATTCAATAAATGCA
MYB21-F	GGGGTACCAATATGCGAATTAGCCTCTCACC
MYB21-R	ACGCGTCGACTATTTTTTTTGGTAGTTTGCGTTGCC
MYB24-F	GGGGTACCCTCACTTTCACAACTCTCCCTTTC
MYB24-R	ACGCGTCGACGAGGCAATCCCATACAGTACTCTG
MYB57-F	GGGGTACCCTATATAGTCGCAAGTCTCAACCC
MYB57-R	ACGCGTCGACCCAGAAGTTACTCAAACACCACC
MYB99-F	GAGAACACGGAGCTCGGTACCATGGGTGGTCGTAAACCATGTTG
MYB99-R	CAGGTCGACTCTAGAGGATCCCTAAACATCGAAACATCCA
ProFLS-F	CCGCTCGAGAATATGCGAATTAGCCTCTCACC
ProFLS-R	CGGGATCCTATTTTTTTTGGTAGTTTGCGTTGCC
GARE1-CHIP-F	TGCGAATTAGCCTCTCACCT
GARE1-CHIP-R	CTCTAAAACAGCAACACCTG
GARE2-CHIP-F	CCTATACTGTAGTTTTTCCT
GARE2-CHIP-R	CCAACCCCATTTAAAGCTAAAG
PY-CHIP-F	CACCCGCCAAAAATATGTAC
PY-CHIP-R	CTCACGAGTTTGGTACCAAG
TA-CHIP-F	CGACGACTTACACATATCAAC
TA-CHIP-R	GTTACGAGTGGTTTTAAGGAG
ACT8-ChIP-F	CCCGCCTATATAAATAGTTCAACAC
ACT8-ChIP-R	GACGACGAGGCAATTCAAAG
MYB21-qRT-F	AGGAGGAAGTAGTGGAGGTT
MYB21-qRT-R	CCGTGGTTGGCGATATAGTT

MYB24-qRT-F	ATGCAAAATGGGGAAATAGGTG
MYB24-qRT-R	AAGATCATCGACGCTCCAATAGTT
FLS1-qRT-F	CCACCGTCATGCGTCAATTACAG
FLS1-qRT-R	TCTCCGCCGAGACCTTCTTTCAA
CHS-qRT-F	GGAGAAGTTCAAGCGCATGTG
CHS-qRT-R	ATGTGACGTTTCCGAATTGTCG
CHI-qRT-F	CTCTCTTACGGTTGCGTTTTCG
CHI-qRT-R	CACCGTTCTTCCCGATGATAGA
F3H-qRT-F	AGGAGGATTCATCGTCTCTAGT
F3H-qRT-R	CACCGTGAGTAGTCTCTGTTTC
F3’H-qRT-F	TTCCTTACCTTCAGGCGGTTATC
F3’H-qRT-R	CGAGAGTGGTGTTGGTGGATG
FLS3-qRT-F	ATGGAATGGTTATCAGAAGGA
FLS3-qRT-R	GACACGGCGGATAGTAAT
UGT78D1-qRT-F	GGCAGAGATAGAAGTTGGA
UGT78D1-qRT-R	GTAGAGATGAGCACAGAGT
UGT78D2-qRT-F	TCCTACATTGACGAATAACCT
UGT78D2-qRT-R	GCCACAGAACCAGAAGAT
UGT79B6-qRT-F	CAAGGTCTCGGTAGAGGTGAAAA
UGT79B6-qRT-R	CGCTCAAGCTCTCCTTCGAA
CCoAOMT-qRT-F	GGTTACTCGCTTCTCACT
CCoAOMT-qRT-R	ACTCACATTTGTCGTTCAC
CAD-qRT-F	TCACTCCTCTGCTTATGC
CAD-qRT-R	TCTCCTCTGTCTCCTTCA
AtF5H-qRT-F	GCCTTAACGGAGTTATTACG

AtF5H-qRT-R	CGATGTCGGATTCTTCAAC
AtMYB99-q-F	TCCGGTGGACTAATTATCTCCG
AtMYB99-q-R	TCTATTGCCAAGGCGAGCAT
AtMYB11-q-F	ACTCCACGGTTACTTCAG
AtMYB11-q-R	CTTCCAGGTCTACGCTTA
AtMYB12-q-F	ATTATTGGAACTCTCATCTCAG
AtMYB12-q-R	TTCATAGCGGACCTACTC
AtMYB111-q-F	ATCCTCACCAAGTATATTCAGA
AtMYB111-q-R	TCCACATCTCAACAATCCA
Tubulin-qRT-F	GAGCCTTACAACGCTACTCTGTCTGTC
Tubulin-qRT-R	ACACCAGACATAGTAGCAGAAATCAAG

Table S2. The quantitative UPLC/Q-TOF MS data of different flavonol derivatives.

	Col-0	*myb21*	*myb24*	*myb57*	*myb21myb24myb57*	*myb11myb12myb111*
**Isorhamnetin-3G-7R**	0.57±0.054	0.35±0.065*	0.55±0.023	0.42±0.069*	0.38±0.018*	0
**Isorhamnetin-3R-7R**	0.22±0.033	0.14±0.026*	0.21±0.032	0.15±0.018*	0.15±0.012*	0
**Q-3RG-7R**	0.08±0.025	0.04±0.032	0.06±0.02	0.06±0.027	0.05±0.005*	0
**K-3RG-7R**	0.56±0.036	0.4±0.047**	0.52±0.052	0.5±0.066	0.37±0.048**	0
**Q-3G-hexose**	0.13±0.044	0.05±0.011**	0.12±0.036	0.08±0.038*	0.06±0.013**	0.11±0.033
**K-3G-hexose**	0.3±0.077	0.17±0.02*	0.3±0.041	0.24±0.033	0.17±0.039*	0.31±0.016
**Q-3G-7R**	0.37±0.069	0.31±0.073	0.35±0.084	0.27±0.091	0.34±0.03	0
**K-3G-7R**	0.52±0.042	0.5±0.061	0.46±0.048*	0.47±0.047	0.42±0.053*	0
**Q-3R-7R**	0.49±0.134	0.35±0.049**	0.45±0.028	0.43±0.089	0.4±0.041*	0
**K-3R-7R**	1.02±0.075	0.79±0.089*	1.03±0.097	0.81±0.21	0.67±0.019**	0
**Total**	4.26±0.233	3.1±0.295*	4.05±0.182	3.43±0.127*	3.01±0.203**	0.42±0.03

	**Col-0**	***MYB21OE1***	***MYB21OE2***	***MYB21OE3***
**Isorhamnetin-3G-7R**	0.63±0.07	0.53±0.038	0.55±0.079	0.59±0.006
**Isorhamnetin-3R-7R**	0.18±0.0102	0.2±0.024	0.22±0.021	0.29±0.028*
**Q-3RG-7R**	0.07±0.0054	0.07±0.011	0.1±0.062	0.13±0.01*
**K-3RG-7R**	0.49±0.0048	0.52±0.056	0.55±0.003**	0.63±0.055
**Q-3G-hexose**	0.11±0.0168	0.15±0.007	0.16±0.016	0.15±0.019
**K-3G-hexose**	0.35±0.0517	0.38±0.008	0.38±0.013	0.52±0.106
**Q-3G-7R**	0.46±0.0008	0.43±0.062	0.43±0.008*	0.52±0.134
**K-3G-7R**	0.54±0.0027	0.78±0.17	0.67±0.001**	0.66±0.044
**Q-3R-7R**	0.25±0.0011	0.36±0.254	0.54±0.005**	0.43±0.02**
**K-3R-7R**	0.88±0.1347	1.01±0.142	0.94±0.003	0.97±0.023
**Total**	3.96±0.034	4.42±0.071*	4.54±0.11*	4.88±0.11**

	**Col-0**	***myb21***	***FLS1OE1myb21***	***FLS1OE2myb21***
**Isorhamnetin-3G-7R**	0.54±0.076	0.35±0.05*	0.46±0.068	0.47±0.062
**Isorhamnetin-3R-7R**	0.16±0.029	0.15±0.0068	0.16±0.034	0.18±0.006
**Q-3RG-7R**	0.07±0.001	0.05±0.0033*	0.05±0.022	0.05±0.021
**K-3RG-7R**	0.51±0.024	0.34±0.0331**	0.55±0.02	0.51±0.067
**Q-3G-hexose**	0.11±0.005	0.08±0.0142*	0.12±0.006	0.11±0.014
**K-3G-hexose**	0.32±0.002	0.2±0.0105*	0.33±0.014	0.33±0.012
**Q-3G-7R**	0.45±0.009	0.17±0.0802**	0.48±0.05	0.45±0.088
**K-3G-7R**	0.56±0.028	0.34±0.0457*	0.6±0.018	0.56±0.08
**Q-3R-7R**	0.19±0.061	0.19±0.0625	0.20±0.261	0.13±0.037
**K-3R-7R**	0.95±0.03	0.83±0.1373	1.02±0.172	1.13±0.133
**Total**	3.86±0.234	2.75±0.478*	3.97±0.399	3.92±0.305

HPLC determination of mean flavonol composition ±SD of Arabidopsis inflorescenses divided into sections. Means±SD, n=3, Asterisks indicate significant differences in flavonol derivatives between WT and other genotypes (***P* < 0.01, **P*< 0.05; Student's *t*-test). The content of different flavonol derivatives with mg/g fresh weitht.

Table S3. The flavonol profiles in methanol-water extracts

Compound Name	v（min）	ESI-MS(m/z)	Fragments(m/z)
Q-3RG-7R	9.29	757	611, 449, 303
K-3RG-7R	10	741	595, 433
Q-3GG	12.02	627	465, 303
Isorhamnetin-3G-7R	13.37	625	463, 317
Q-3G-7R	11.14	611	449, 303
K-3GG	13.78	611	449, 287
Isorhamnetin-3R-7R	16	609	463, 317
K-3G-7R	12.89	595	433, 287
Q-3R-7R	13.23	595	449, 303
K-3R-7R	15.39	579	433, 287
Sinapoyl derivatives	10	369	207
